# Anticipation strategies of motor control in children and adolescents with cerebellar pathologies and typical development: a dual task paradigm

**DOI:** 10.3389/fneur.2026.1830073

**Published:** 2026-06-24

**Authors:** S. Gazzellini, E. Lovardi, T. Schirinzi, E.S. Marotta, S. Summa, C. Smargiassi, S. Mazzoleni, F. Benso, E. Castelli, D. Lettori, G. Vasco

**Affiliations:** 1 Neuroscience Clinic Area, Neurorehabilitation Research Area, Bambino Gesù Children’s Hospital, IRCCS, Rome, Italy; 2 Neurology Unit, Department of Systems Medicine, University of Rome “Tor Vergata”, Rome, Italy; 3 The Link Centre, Plumpton, United Kingdom; 4 Department of Electrical and Information Engineering, Polytechnic University of Bari, Bari, Italy; 5 IMT School for Advanced Studies of Lucca, Lucca, Italy; 6 Department of Psychology and Cognitive Sciences, University of Trento, Rovereto, Italy

**Keywords:** anticipation, cerebellum, dual task, executive functions, motor control, pediatric

## Abstract

**Background:**

Besides ataxia, cerebellar pathologies are often associated with neuropsychological deficits, particularly in executive attention. The cerebellum is considered a key hub of the predictive brain network, interacting with prefrontal and parietal areas through the basal ganglia. Anticipating the sensory consequences of motor actions and comparing them with actual feedback supports feedforward control, improving coordination, processing speed, and reducing errors. It also contributes to integrating subcortical–cortical networks during dual-task conditions. We hypothesized that impaired anticipatory strategies contribute to motor control deficits in cerebellar patients. Specifically, fine upper-limb motor control would worsen when combined with concurrent neuropsychological and cerebellar tasks (verbal anticipation and timing), compared with a working memory task or a single motor task. Therefore, we aimed to investigate dual-task abilities in cerebellar patients.

**Method:**

In total, 24 participants affected by cerebellar pathologies (mean age was 13y 1 m; 16 females) and 24 participants with typical development (mean age was 13y 1 m; 14 females) were required to execute a single visuo-motor precision task (single task condition) alone and with concurrent verbal anticipation, verbal timing, and verbal nBack tasks. Execution time and accuracy were analyzed as dependent variables combined in a single index [Inverse Efficacy, IE = RT/(1-pE)] and separately.

**Results:**

The results showed that the dual-task condition negatively affects the fine motor control of the upper limb in the two groups. A significant negative effect of the anticipation task was found for both the cerebellar and control groups. The cerebellar group presented a significant negative effect on the nBack task as well.

**Discussion:**

The results are consistent with the models describing the cerebellum as a key hub in anticipatory control, probably through predictive network synchronization. The anticipatory control is a damaged component in the control deficit of cerebellar patients. Finally, the cerebellar role in synchronizing the different cerebral networks involved in dual-task processing is strengthened. This study underlines the crucial role of anticipatory and feedforward mechanisms in motor control of both typically developed children and those with cerebellar pathologies and defines the importance of predictive neuropsychological skills in cognitive and motor fluidity.

## Introduction

Pediatric ataxias constitute a broad and highly heterogeneous group of rare neurological disorders characterized by impaired balance, coordination, and motor control resulting from dysfunction of the cerebellum and its afferent and efferent pathways. The cerebellum plays a central role not only in motor coordination and postural control but also in motor learning, timing, prediction, and higher cognitive and affective functions. Consequently, cerebellar dysfunction in childhood has a pervasive impact on both motor and non-motor development, with long-term consequences on autonomy, social participation, and quality of life (1–7). Clinically, pediatric ataxias present with gait and stance instability, limb dysmetria, intention tremor, dysarthria, and oculomotor abnormalities such as nystagmus, saccadic dysmetria, and impaired smooth pursuit (4). These core features are frequently accompanied by extracerebellar manifestations, including peripheral neuropathy, pyramidal signs, extrapyramidal features, epilepsy, cognitive impairment, and behavioral disturbances, reflecting the multisystem nature of many ataxic disorders (8, 9). When onset occurs during childhood, these manifestations interfere with critical phases of neurodevelopment, motor acquisition, and learning processes, often leading to cumulative disability over time and placing a substantial burden on families and caregivers (10–12). From an etiological and nosological perspective, pediatric ataxias may be classified as non-progressive or progressive, and as isolated cerebellar forms or syndromic conditions with multisystem involvement. The majority are of genetic origin, although immune-mediated, metabolic, mitochondrial, and acquired causes are increasingly recognized. To date, more than 100 genes have been implicated, encoding proteins involved in mitochondrial homeostasis, oxidative stress response, DNA repair mechanisms, synaptic transmission, ion channel function, and protein quality control (8). This remarkable genetic and molecular heterogeneity translates into a wide spectrum of clinical phenotypes, disease trajectories, and rates of progression (2). Among the most prevalent genetic pediatric ataxias are Friedreich ataxia (FRDA), ataxia-telangiectasia (A-T), and the spinocerebellar ataxias (SCAs). In contrast, conditions such as Joubert syndrome and related disorders are typically non-progressive and associated with cerebellar hypoplasia or dysplasia, yet still result in significant lifelong disability (13).

Typically, cerebellar pathologies manifest with ataxia, but also with alterations in cognitive performance, deficits in executive functions, impairments in linguistic and visuospatial abilities (14, 15), affective disturbances, and mood alterations (see (16), for developmental evidence). These neuropsychological alterations have been conceptualized as the *Cerebellar Cognitive–Affective Syndrome* (CCAS) or the *Dysmetria of Thought* hypothesis (5, 17–19).

From a maturational perspective, it has been observed that the developmental trajectory of the posterior lobe of the cerebellum follows a temporal pattern like that of the prefrontal cortex. Both regions are considered ontogenetically late developing, in the sense that they reach full maturation later in childhood, approximately around 9 years of age (16). The mechanism through which the cerebellum operates and its key role within prefrontal circuits have been theorized in the concept of the *Universal Cerebellar Transform*: the cerebellum integrates its internal representations with external stimuli and implicitly generates responses aimed at modulating performance in accordance with context (5, 18). Further evidence suggests that cerebellar processing mostly depends on a supervised learning mode (20, 21). Moreover, sequence detection would represent a cerebellar operational mode: the cerebellum detects and simulates repetitive spatial or temporal patterns of perceptual events, generating internal models employed to make predictions of future events (22).

The focus of the present work is on the relationship between cerebellar functions of anticipation/prediction and motor control. Indeed, in classifying motor control modes, Patla (23) underlined the relevance of anticipatory strategies, in association with predictive and reactive strategies. Consistently, after studying adult patients with cerebellar degeneration, in which the cerebellar cortex was diffusely affected, Olivito and colleagues (24) showed that the cerebellum plays a key role in executive control of action inhibition, both proactive and reactive control. Ivry and colleagues described a model of the “predictive brain,” involving the cerebellum as a crucial node in a PFC-IPL-Cbllm network (i.e., prefrontal-inferior parietal cortex and cerebellum) throughout the thalamus and basal ganglia. In particular, the cerebellar role would consist of synchronizing the timing lag between the motor output representation into the prefrontal area and the efference copy simultaneously sent to parietal lobules (25). The efference copy is a copy of the motor command sent back from frontal to parietal areas in a top-down modulation, which can pre-activate all the sensory perceptions (visual, proprioceptive, haptic, tactile, and auditory) associated with the motor gesture (26–28). The predictive brain network achieves motor control in a feedforward modality by comparing such sensory expectancies with actual sensory feedback (29). Sensory expectancies, built on prior experience, ensure the fluid operation of cognitive and motor functions, decreasing individual behavior variability and allowing for forward-anticipatory rather than feedback-reactive control (25). Moreover, attention mechanisms contribute to strictly linking expectancies and the environment, decreasing the probability of being distracted by task-irrelevant stimuli and creating a helpful future-oriented brain state.

Besides forward models, many computational models of motor control also “incorporate inverse models that invert the computation of the system in that they provide the motor command which will result in a desired end-state (e.g., a particular sensory state) given a particular current state” (26, 28). Donnarumma and colleagues (30) implemented a computational model of action observation, relying on an internal generative model that generates predictions of the next sensory state. The model is based on a hypothesis-testing process that drives anticipatory eye movements (i.e., saccades) toward the most informative parts of the visual scene.

From a clinical perspective, a neuropsychological study by Butti and colleagues (31) conducted on children and adolescents supports the presence of a specific deficit in patients who survived tumors affecting cerebellar areas, namely in forming or relying on contextual priors during action observation. The role of the cerebellum in action prediction cannot be fully explained by motor simulation mechanisms alone and should instead be framed within a predictive coding account, in which the cerebellum exerts a modulatory function by providing contextual priors that guide the selection of the most likely outcome of a given action.

In line with this view, a virtual reality–based rehabilitation program was developed for children with cerebellar malformations, aiming to enhance social predictive abilities by training the use of context-based expectations (32). The study demonstrates the clinical effectiveness of virtual reality training in patients with cerebellar lesions.

Oldrati and colleagues (33) tested the use of anodal cerebellar transcranial Direct Current Stimulation (ctDCS) as a method to improve contextual prediction of others’ actions in a sample of adolescents and young adults with congenital, non-progressive cerebellar malformations. The results confirmed a facilitative effect of anodal ctDCS compared with sham stimulation on action prediction in moderately informative contexts.

The present work was aimed at studying the weight of anticipatory strategies in upper limb motor control of young healthy participants and young patients with cerebellar pathologies, which cause motor deficits. We hypothesize that engaging participants in a verbal anticipation task will negatively affect the anticipation component of motor control and hence negatively interfere with upper limb motor execution more than a memory control task. From an anatomo-functional perspective, the nBack working memory task relies mainly on prefrontal dorsolateral structures (34, 35). The anticipation function involves a cerebellum-parietal–frontal network [and basal ganglia: (36, 37)] in which the cerebellar contribution to synchronize cortical activity is preeminent. Therefore, we hypothesize that the upper-limb motor control of participants with cerebellar disease and dysfunction would be more affected by a simultaneous anticipation cognitive requirement with respect to a working memory task and a basal, single-task motor condition. We hypothesize a reduced, but nevertheless present, anticipation effect in typical development participants with respect to the clinical group, if the anticipation strategy is relevant even in typical motor control. Finally, timing is a cerebellar-driven function as well (36, 38, 39). We hypothesize a similar effect in the width of timing on motor control as the anticipation task, both in the group with cerebellar pathology and that with typical development.

## Methods

### Participants

Overall, n. 48 participants have been recruited in the study, both children and adolescents, grouped into a control and an experimental sample (see Table 1). In the experimental Cerebellar group, we enrolled 24 patients diagnosed with cerebellar pathologies (mean age = 13.1 years, SD = 3.3 years, range 8.3–17.11): 8 were male and 16 were female. The patients were recruited from the Pediatric Neurorehabilitation Unit of “Bambino Gesù” Children’s Hospital of Rome (Italy) and presented the following cerebellar pathologies: Friedreich’s ataxia (*n* = 8), genetically determined cerebellar ataxia from COQ8A/ADCK3 mutation (*n* = 5), and neurological outcomes from neurosurgical removal of posterior cranial fossa (PCF) tumors (*n* = 5), including pilocytic astrocytoma and medulloblastoma. The group also included patients with Joubert syndrome (*n* = 2), ataxia-telangiectasia (*n* = 1), SQSTM-related ataxia with vermis atrophy (*n* = 1), ITPR1-mutation with cerebellar atrophy (*n* = 1), and one case of cerebellar ataxia Not Other Specified (NOS) (*n* = 1). Progressive pathologies have been considered: Friedreich ataxia, ataxia telangiectasia, STQSM1 mutation (n. 10). Non-Progressive pathologies include Joubert syndrome, Posterior cranial fossa tumors, ITPR1 mutation, ADCK3 mutation, and cerebellar ataxia “of undetermined nature” (n. 14). Each patient has been evaluated with the SARA, Scale for the Assessment and Rating of Ataxia (6), at the moment of the experimental session: the higher SARA score, the higher the level of ataxia and motor dysfunction.

**Table 1:** Demographic and clinical characteristics of the cerebellar and TD groups.

Participant *n*	Group	Sex	Age (y.m)	IQ (percentile)	Attention (percent)	Diagnosis	Progression	SARA score
1.1	Cerebellar	F	16.0	7°	38°	Joubert syndrome	Non-progressive	5
1.2	Cerebellar	F	14.6	14°	38°	*COQ8A*-Ataxia (*ADCK3)*	Non-progressive	9
1.3	Cerebellar	F	13.10	0.1°	1°	Joubert syndrome	Non-progressive	16.5
1.4	Cerebellar	F	16.5	50°	38°	Friedreich’s Ataxia	Progressive	9
1.5	Cerebellar	F	17.11	0.1°	1°	Ataxia-Telangiectasia	Progressive	24
1.6	Cerebellar	M	8.6	15°	3.5°	*COQ8A*-Ataxia (*ADCK3)*	Non-progressive	15
1.7	Cerebellar	M	11.2	37.5°	1°	*COQ8A*-Ataxia (*ADCK3)*	Non-progressive	13.5
1.8	Cerebellar	M	14.11	2.4°	8°	PCF tumor (Cerebellar peduncle)	Non-progressive	12
1.9	Cerebellar	F	16.2	37°	38°	*SQSTM* -Ataxia (Vermis Atrophy)	Progressive	8.5
1.10	Cerebellar	F	9.3	92.5°	3.5°	Friedreich’s Ataxia	Progressive	9
1.11	Cerebellar	F	10.3	63°	38°	Friedreich’s Ataxia	Progressive	19.5
1.12	Cerebellar	F	14.7	39°	3.5°	PCF Pilocytic Astrocytoma	Non-progressive	7
1.13	Cerebellar	F	8.3	0.3°	1°	*ITPR1*-Ataxia (Cerebellar Atrophy)	Non-progressive	11
1.14	Cerebellar	F	9.11	37.5°	63°	*COQ8A*-Ataxia (*ADCK3)*	Non-progressive	8.5
1.15	Cerebellar	M	8.3	92.5°	8°	*COQ8A*-Ataxia *(ADCK3)*	Non-progressive	0.5
1.16	Cerebellar	M	8.5	>95°	18°	Friedreich’s Ataxia	Progressive	10.5
1.17	Cerebellar	M	11.6	25°	16°	PCF Pilocytic Astrocytoma (4th Ventricle, R Cerebellar Peduncle)	Non-progressive	17.5
1.18	Cerebellar	F	14.1	5°	8°	Cerebellar Ataxia, NOS	Non-progressive	5.5
1.19	Cerebellar	F	15.3	37.5°	38°	Friedreich’s Ataxia	Progressive	15.5
1.20	Cerebellar	M	17,5	83°	38°	Friedreich’s Ataxia	Progressive	12
1.21	Cerebellar	M	13.7	11°	38°	PCF Astrocytoma	Non-progressive	16
1.22	Cerebellar	F	13.3	26.5°	3.5°	PCF Medulloblastoma (Grade IV)	Non-progressive	8.5
1.23	Cerebellar	F	16.9	92°	38°	Friedreich’s Ataxia	Progressive	6
1.24	Cerebellar	F	16.2	74°	38°	Friedreich’s Ataxia	Progressive	8
			Mean: 13.1 SD: 3.3	Median: 37.25	Median: 17			

Participant *n*	Group	Sex	Age (y.m)	IQ (percentile)	Attention (percent)
2.1	TD	F	9.4	>95°	38°
2.2	TD	F	9.11	82°	8°
2.3	TD	F	8.6	85°	63°
2.4	TD	F	12.6	15°	3,5°
2.5	TD	F	12.4	89°	38°
2.6	TD	F	14.7	42.5°	38°
2.7	TD	F	15.9	60°	3,5°
2.8	TD	F	16.3	91°	3,5°
2.9	TD	F	16.4	60°	38°
2.10	TD	M	14.11	92°	38°
2.11	TD	M	11	75°	38°
2.12	TD	F	17.6	50°	38°
2.13	TD	M	10.10	88°	38°
2.14	TD	F	11.8	47°	38°
2.15	TD	M	15	50°	38°
2.16	TD	M	14.1	37°	8°
2.17	TD	M	14.1	16°	8°
2.18	TD	M	13.1	50°	38°
2.19	TD	M	12.7	38°	38°
2.20	TD	F	15.5	37°	38°
2.21	TD	M	14.3	74.5°	38°
2.22	TD	M	16.8	37.5°	1°
2.23	TD	F	14.0	74.5°	38°
2.24	TD	F	14.8	4.95°	8°
			Mean: 13.1 SD: 2.11	Median: 55	Median: 38

The table reports sex, age, IQ, and attention percentiles for both groups, with diagnosis, disease progression, and SARA scores for the cerebellar group.

The control group consisted of 24 typically developing (TD) children and adolescents, the mean age was 13.1 years, the standard deviation (SD) was 2.11 years, the range was 8.6–17.6, and there were 10 male and 14 female participants.

Cognitive level (IQ percentile) for the Cerebellar and TD group was assessed using age-appropriate standardized instruments tailored to the patients’ clinical conditions. Non-verbal intelligence was evaluated using the Leiter International Performance Scale-Third Edition [Leiter-III; (40)], Raven’s Colored Progressive Matrices [CPM; (41, 42)], or Standard Progressive Matrices [SPM; (41, 43)]. General cognitive functioning was measured via the Wechsler Intelligence Scale for Children-Fourth Edition [WISC-IV; (44)] or the Wechsler Preschool and Primary Scale of Intelligence-Third Edition [WPPSI-III; (45)]. Additionally, sustained attention abilities were evaluated through the “Auditory Attention” subtest of the NEPSY-II developmental neuropsychological battery (46, 47). The percentiles of IQ and attention level for each participant are reported in Table 1. The Cerebellar group exhibited a lower median IQ percentile of 37.25, and a lower median Auditory Attention percentile of 17; with respect to the TD group, the median IQ percentile was 55, and the median Auditory Attention percentile was 38.

The testing protocol consisted of two sessions separated by a rest period. The first session included IQ and attention assessments with a minimum duration of 15 min. To prevent fatigue, the Cerebellar group completed longer IQ assessments on a separate day. The second session comprised the experimental tasks, lasting 30 min overall.

Written informed consent was obtained from the minors’ legal guardian for the publication of any potentially identifiable data included in this article. The study was conducted in accordance with the Declaration of Helsinki. The procedure was approved by the Ethics Committee of Bambino Gesù Children’s Hospital (Rome, Italy).

### Procedure

The procedure involved the administration of an experimental dual-task paradigm in which two activities are required to be performed simultaneously. In the case of the present study, the former and basal performance requires upper limb fine motor skills. The latter are cognitive tasks requiring anticipation, memory, and timing processes, in a randomized order of administration. By comparing the performance of each patient during the basal motor condition (Single Task, ST) and the same during the Dual Task (DT) conditions, it is possible to determine if the two tasks, motor and cognitive, compete for the same resources within the nervous system and what are the consequences of their actions in the patient group with Cerebellar pathologies and in the TD group. The motor task is taken from Nepsy 2 [Neuropsychological Assessment Battery, and in particular the “Visuomotor Precision Task” of the Sensorimotor Functions domain: test–retest reliability *r* = 0.60–0.81, internal consistency *r* = 0.50–0.93, in clinical samples *r* values >0.80; (46, 47)]. The task requires a person to draw a line within a path as quickly and accurately as possible. We recorded the speed and accuracy of motor execution. Two paths were administered, with two levels of motor complexity (see Appendix). The time execution (in ms) and number of errors of the two paths have been summed to get the overall score of performance. The routes administered were divided into segments for error counting. Errors were defined as follows: (1) boundary violations: any instance where the drawn line crossed or overlapped with the peripheral lines of the path; (2) pen lifts: any instance where the participant lifted the pencil from the paper before completing the trial.

The administration order of the following four experimental conditions was randomized across participants (both TD and patients). Consequently, each task condition appeared six times in each ordinal position for the group. The testing protocol consisted of two sessions separated by a rest period. The first session included IQ and attention assessments with a minimum duration of 15 min. To prevent fatigue, the Cerebellar group completed longer IQ assessments on a separate day. The second session comprised the experimental tasks, lasting 30 min overall.

The administration order of the following four experimental conditions was randomized across participants (both TD and patients). Consequently, each task condition appeared six times in each ordinal position for the group.

In the Single Task (ST) condition, only the motor task was performed to establish a baseline. In the Dual Task (DT) conditions, the motor task was performed concurrently with one of three cognitive tasks.

**Figure 1:**
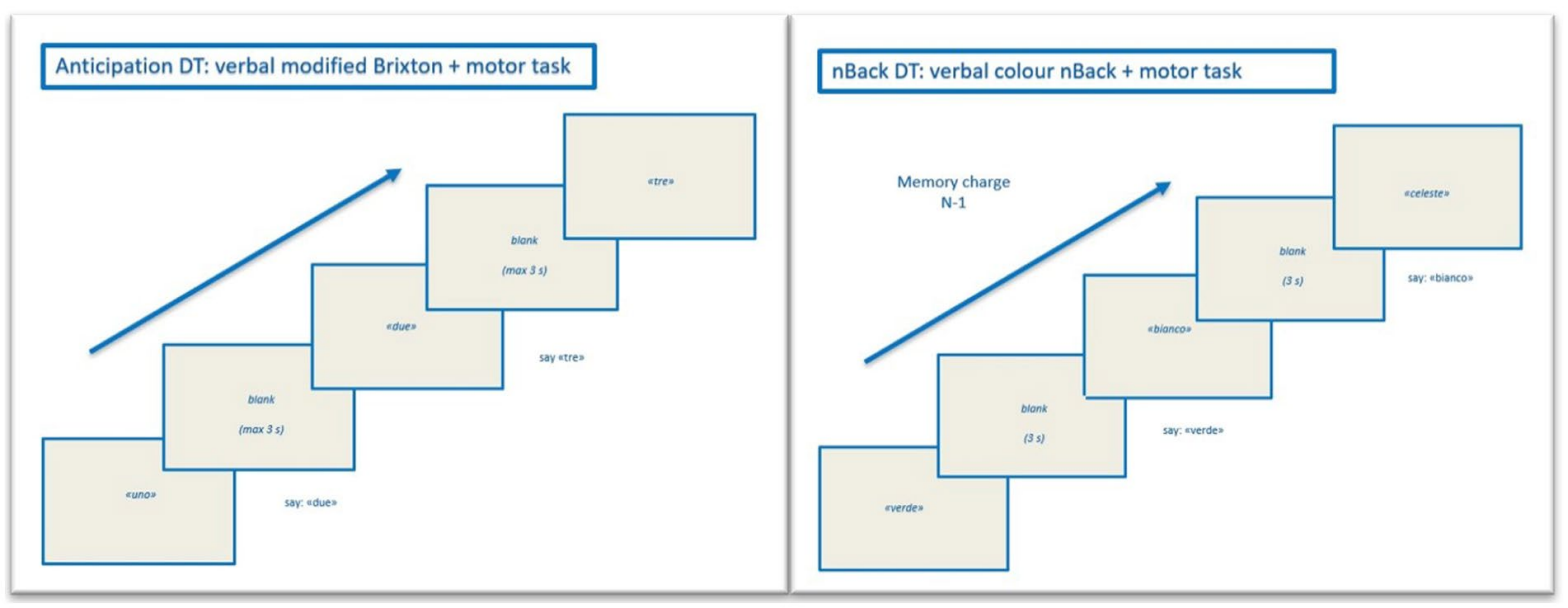
Procedure of the Anticipation task and nBack task.

*Anticipation DT*: The experiment was programmed using E-Prime 3.0 software (48) and consisted of a verbal adaptation of the Brixton Spatial Anticipation Test (49), designed to allow for dual-task presentation alongside a motor task. The procedure comprised a session of 56 potential trials, each structured as follows (see Figure 1). *Stimuli*: the presentation of auditory stimulus lasting a maximum of 3,000 ms. Participants were given the following instructions: “You will hear a voice reading a number. You must guess what the subsequent number will be. Do not be concerned if you get the first number wrong.” The task began with a standard numerical sequence (1, 2, 3, 4, 5, 6…) and progressed to more complex patterns (e.g., 10, 5, 10… or 8, 9, 8…), following the structure of the original visuo-spatial Brixton. The subsequent stimulus in the sequence served as an informative feedback signal, enabling participants to verify the correctness of their preceding anticipation. *Inter-trial interval*: the maximum duration was 3,000 ms, depending on response production. Although the task consisted of 56 numerical stimuli, the end of the test was determined by the completion of the concurrent motor task. Consequently, the number of trials performed varied according to individual motor execution speed. The number of errors was manually recorded by the researcher, and cognitive performance was calculated as the error percentage relative to the trials actually completed.

*NBack DT*: The experiment was programmed using E-Prime software and consisted of a working memory task labeled n-Back DT, designed for dual-task presentation alongside a motor task. The procedure was organized into the following stages. *Stimuli:* the presentation of a randomized sequence of color names emitted by a computerized voice. Participants were given the following instructions: “You will hear a voice reading the names of colors. Every time you hear a color, you must state the name of the previous color. E.g., WHITE–GREEN (now you must say WHITE).” Participants were required to verbally report the stimulus presented immediately prior to the current one (1-back: Figure 1). *Inter-trial interval*: the maximum duration was 3,000 ms, depending on response production. In this case as well, the end of the test was determined by the completion of the concurrent motor task. Consequently, the number of trials performed varied according to individual motor execution speed. The number of errors was manually recorded by the researcher, and cognitive performance was calculated as the error percentage relative to the trials actually completed.

*Timing DT*: The procedure was organized into the following stages. *Synchronization Phase*: the presentation of a rhythmic acoustic signal emitted by a computerized metronome set at 50 BPM. Participants were required to vocalize the syllable “*la*” in precise synchronization with the beat. *Task Logic*: the concurrent motor task commenced only after the participant had successfully synchronized with the metronome’s tempo. The end of the task was determined by the end of the motor task.

The concurrent tasks (Anticipation, nBack, and Timing) utilized exclusively auditory-verbal input and output modes, since they were specifically chosen to avoid taxing the visual and motor channels, which remained reserved for the primary motor task.

### Statistical analyses

As the primary dependent variable, we employed the Inverse Efficiency index [IE; (50, 51)]. Since some subjects might show the effects of interest in response speed while others might show them in accuracy, we combined these scores using the composite measure of IE to account for the speed-accuracy trade-off. IE combines execution time and error rate*: IE = RT/(1 − pE)*, where *RT* is the overall execution time (i.e., the sum of the durations of the two trials) and *pE* is the proportion of errors. Thus, higher IE values indicate poorer performance. Normality was assessed on model residuals using both the Shapiro–Wilk test and Q–Q plots. Homogeneity of variances between groups was assessed using Levene’s test, which indicated some heterogeneity (*p* = 0.015). However, given the relatively balanced design and the absence of extreme variance differences, this violation was considered moderate. Sphericity of the within-subject factor was assessed using Mauchly’s test. Overall, considering that mixed-design ANOVA is well-established as robust to moderate violations of normality and homogeneity, two-way mixed ANOVAs (Group X Condition: 2 × 4) were conducted, with Group as a between-subjects factor and Condition as a within-subjects factor. IE, RT, and percentage of errors were used as dependent variables. Tukey’s HSD *post hoc* test was applied when significant main effects were observed. Finally, the SARA score was included as a covariate in the ANOVA model.

## Results

**Figure 2:**
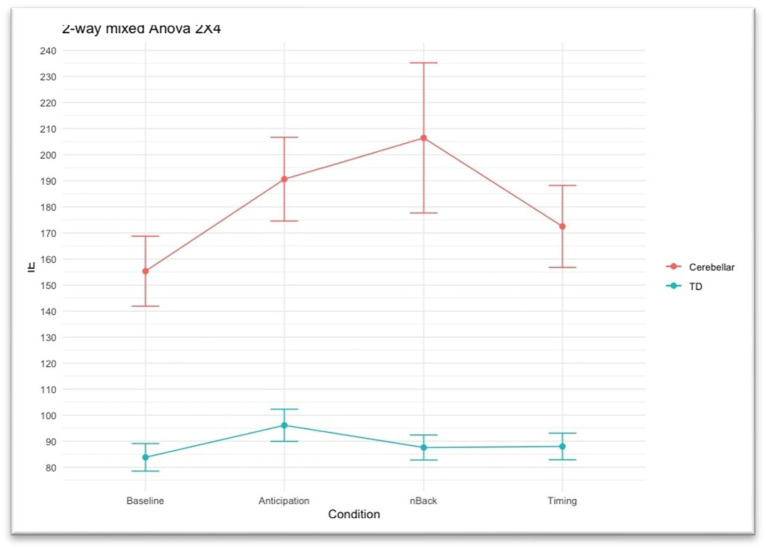
Group X condition interaction: IE mean values and standard errors.

The two-way mixed ANOVA (Group X Condition: 2 × 4) with Group as a between factor and Condition as a within factor (mixed design), on the IE index as the dependent variable, reported the following significant main factors: Group [*F*(1,46) = 27.7, *p* < 0.001, η^2^ = 0.31], Condition [*F*(3,138) = 4.8, *p* < 0.01, η^2^ = 0.02]. The interaction Group X Condition [*F*(3,138) = 3.02, *p* < 0.05, η^2^ = 0.02] was significant (see Figure 2). The mean values and SDs of the IE index are shown in Table 2.

**Table 2:** IE mean values and standard deviations for each condition and group (without any correction).

dep var: IE = RT/(1-pE)	Single task (baseline) mean (SD)	Anticipation DT	nBack DT	Timing DT
Typical development	83.8 (26.1)	96.1 (30.3)	87.6 (23.6)	88 (25)
Cerebellar	155.3 (65.8)	190.6 (78.6)	206.4 (141.1)	172.5 (76)

DT, Dual task; SD, Standard deviation.

**Figure 3:**
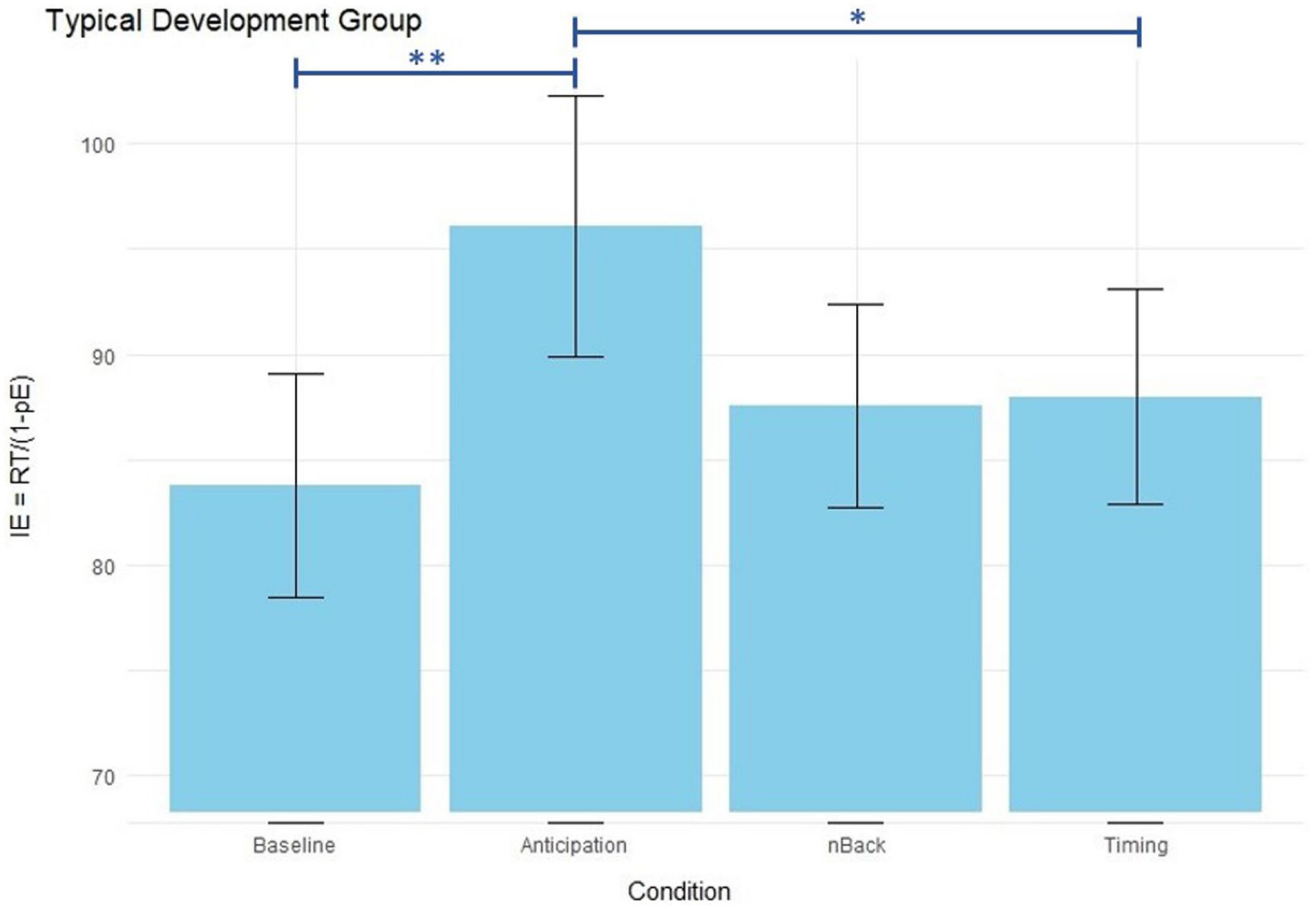
TD group: IE mean values and standard errors. *p < 0.05, **p < 0.01, Tukey’s HSD *post hoc*.

As far as the TD group is concerned, the mean IE values were highest in the Anticipation DT condition (96.1, SD 30.3), followed by 88 (25) in Timing DT, 87.6 (23.6) in nBack DT, and the minimum value was found in the ST condition (83.8, SD 26.1), as expected (see Figures 2, 3). Condition was a significant factor even for the ANOVA carried out on the TD group only [*F*(3,69) = 3.7, p < 0.05 η^2^ = 0.14].

Tukey’s HSD post-hoc test revealed significantly larger IE values in the Anticipation DT condition with respect to ST [*t*(23) = 2.6, *p* < 0.01] and Timing DT [*t*(23) = 2.3, *p* < 0.05]. The conditions ST, nBack DT, and Timing DT did not differ from each other for IE value.

**Figure 4:**
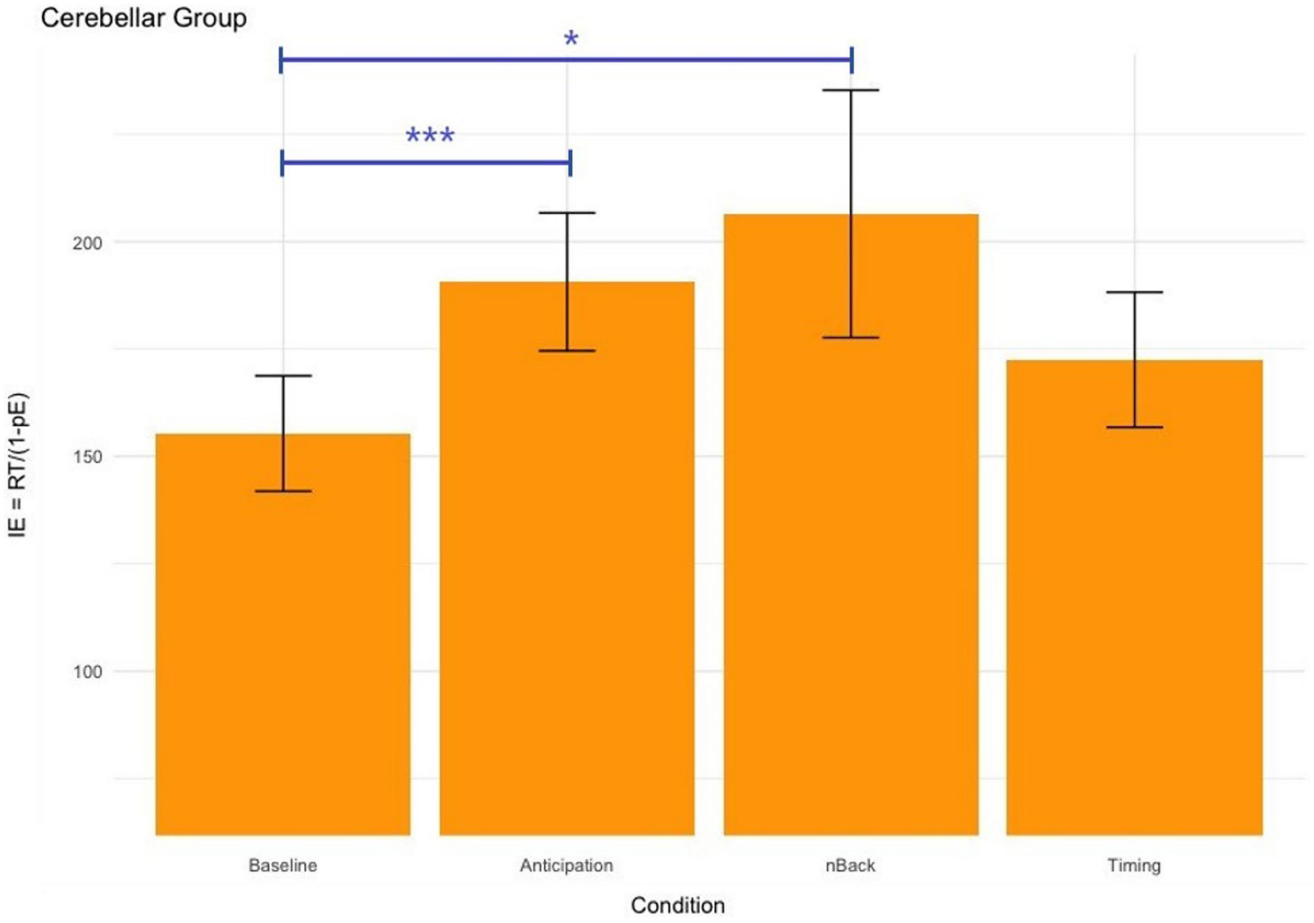
Cerebellar group: IE mean values and standard errors. *p < 0.05, **p < 0.01, Tukey’s HSD post hoc.

On the other hand, the Cerebellar group performed with overall higher IE values. Student’s *t*-tests when comparing IE values between TD and Cerebellar groups for each condition were as follows: Baseline [*t*(46) = 4.9, *p* < 0.001], Anticipation [*t*(46) = 5.5, *p* < 0.001], Timing [*t*(46) = 5.1, *p* < 0.001], and nBack [*t*(46) = 4.1, *p* < 0.001]. Moreover, the Cerebellar group showed larger behavioral variation (i.e., SD) than the TD group. Average and standard deviation values for the Cerebellar group were 190.6 and 78.6 in the Anticipation DT condition, 206.4 and 141.1 in nBack DT, 172.5 and 77 in Timing DT, and the minimum value was found again in the ST condition: 155.3 and 65.8 (see Table 2; Figures 2, 4). Condition was a significant factor even in the ANOVA carried out on the Cerebellar group only [*F*(3,69) = 3.9, *p* < 0.05, η^2^ = 0.14].

Tukey’s HSD post-hoc test (Figure 4) revealed a significantly larger IE values in the Anticipation DT condition with respect to ST [*t*(23) = 5.9, *p* < 0.001], and in nBack DT condition with respect to ST as well [*t*(23) = 2.4, *p* < 0.05]. The conditions ST and Timing DT did not differ from each other for IE value.

We report the same analysis structure using RTs and the percentage of errors as separate dependent variables. Mean values and standard deviations for each Condition and Group are reported in Table 3. The mixed ANOVA (Group X Condition: 2 × 4) on RTs reported the following significant main effects: Group [*F*(1,46) = 22.3, *p* < 0.001, η^2^ = 0.27], Condition [*F*(3,138) = 9.7, *p* < 0.001, η^2^ = 0.05]. The interaction Group X Condition [*F*(3,138) = 4.5, *p* < 0.01, η^2^ = 0.02] was significant. Tukey’s HSD post-hoc on TD group showed the following mean values in ms (ST = 80,082, Anticipation DT = 92,384, Timing DT = 84,283, nBack = 83,467), with significantly higher mean values for the Anticipation DT condition with respect to ST [*t*(23) = 2.7, *p* < 0.05], and Timing DT [*t*(23) = 2.3, *p* < 0.05]. The other contrasts did not differ from each other. The same analyses on the Cerebellar group showed the following mean values in ms (ST = 113,085, Anticipation DT = 144,158, Timing DT = 127,053, nBack = 148,242) with significant differences in all the contrasts, but Anticipation vs. nBack.

**Table 3:** RTs and percentage of error, mean values, and standard deviations for each condition and group.

	Single task (baseline)		Anticipation DT		nBack DT		Timing DT	
	RTs mean (SD): s	% Errors	RTs	% Errors	RTs	% Errors	RTs	% Errors
Typical development	80.082 (25.682)	4.7 (3.1)	92.384 (28.537)	3.7 (2.2)	83.467 (23.684)	4.9 (3.1)	84.283 (24.747)	4.4 (2.5)
Cerebellar	113.085 (37.633)	22.5 (20.1)	144.158 (47.981)	19.5 (20.1)	148.242 (65.504)	20.1 (18.6)	127.053 (47.393)	22.2 (18)

DT, Dual task; SD, Standard deviation.

The mixed ANOVA (Group X Condition: 2 × 4) on the percentage of Errors showed the significant main factor Group (*F*(1,46) = 18.9, *p* < 0.001, η^2^ = 0.29). The factor Condition (*F*(3,138) = 2.3, n.s.) and the interaction Group X Condition (*F*(3,138) = 1.3, n.s.) were not significant. Mean values for the significant factor Group were as follows: 4.4% for the TD group and 21.1% for the Cerebellar group.

**Figure 5:**
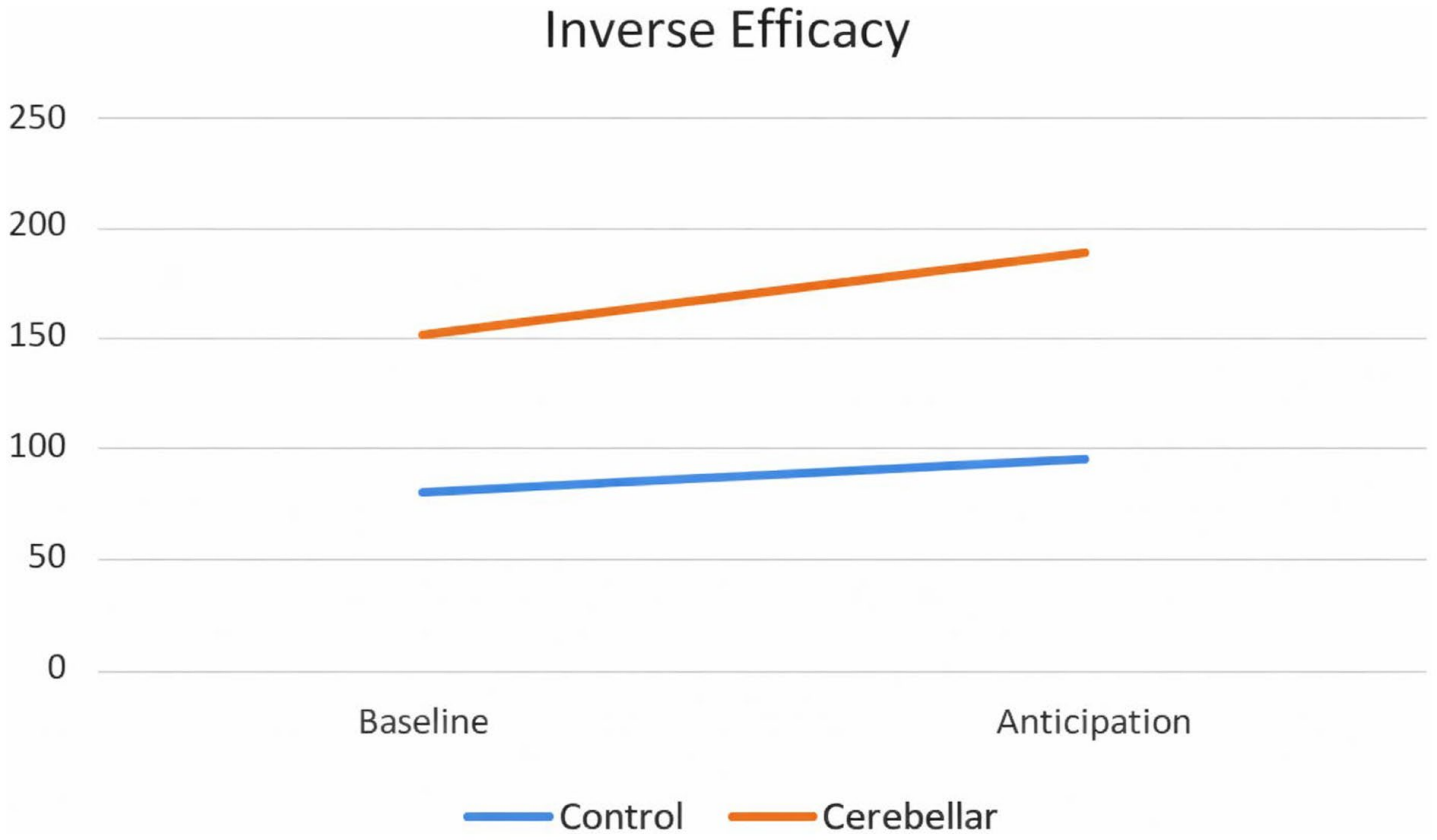
Anticipation effect X group significant interaction on IE.

Coming back to IE as the dependent variable, we further explored possible differences in the Anticipation effect (Anticipation DT vs. ST) between the two groups of young participants with Typical Development and persons with Cerebellar disease. A mixed ANOVA with Condition (2 levels: Anticipation DT, ST) as the within-subjects factor and Group as the between-subjects factor showed the following significant main effects and interaction: Condition (*F*(1,46) = 39.3, *p* < 0.001), Group (*F*(1,46) = 29.0, *p* < 0.001), and interaction Group X Condition (*F*(1,46) = 9.2, *p* < 0.01; Figure 5). *Post hoc* comparisons showed a significant difference in Anticipation DT vs. ST both for TD (*t*(24) = 2.6, *p* < 0.05) and Cerebellar group (*t*(24) = 5.9, *p* < 0.001), and the significant main factors interaction proved a larger Anticipation effect (Anticipation DT > ST) of the Cerebellar group with respect to the TD group. Interference percentages (Anticipation effect) have been calculated for each participant, following the formula (Ant. IE/Base. IE)*100)-100). Mean values of interference percentages were +17.5 for the TD group and +24.1 for the Cerebellar group. T-Student statistics were not significant (*t*(46) = 0.86, n.s.), even if mean values are in line with greater interference percentage for patients with cerebellar pathologies.

The SARA is a scale for the assessment and rating of ataxia (6). The main factor Condition remained significant (*F*(3,74) = 2.93, *p* < 0.05) even when the cerebellar patients’ SARA score was introduced as a covariate factor in the ANOVA model. SARA score was also a significant covariate predictor on the IE index of ataxic persons, as we analyzed in the model (*F*(18,74) = 7.47, *p* < 0.001). Participants with cerebellar pathologies were moreover classified into High and Low SARA scores according to the group median SARA value of 9. This resulted in n. 12 participants in the Low group (SARA score ≤9), and n. 12 participants in the High group (SARA score >9). The mixed ANOVA on the factors SARA_grade (low, high) and Condition revealed the statistical significance of both factors: SARA_grade (*F*(1,22) = 6.86, *p* < 0.05, η^2^ = 0.18), Condition (*F*(3,66) = 4.26, *p* < 0.01 η^2^ = 0.05), and a significant SARA_grade X Condition interaction (*F*(3,66) = 3.07, *p* < 0.05 η^2^ = 0.04). After running *post hoc* analyses, we found that while a significant Anticipation DT vs. ST difference was present for all the cerebellar participants in both High and Low SARA_grade groups (mean IE values 222.5 *Vs* 186.5, respectively, (*t*(11) = 4.12, *p* < 0.01) for High, and 158.4. vs. 124.2, for Low SARA_score (*t*(11) = 4.1, *p* < 0.01), the difference between nBack vs. ST was significant only for the more severe cerebellar participants belonging to the High SARA-Grade group (mean 273.9. vs. 186.5, respectively, (*t*(11) = 2.2, *p* < 0.05). In the Low SARA-Grade group, Anticipation DT effect was significantly larger than nBack DT (mean 158.4. vs. 139, respectively, (*t*(11) = 2.31, *p* < 0.05). None of the other comparisons were significant, e.g., for the Timing task.

Patients with Cerebellar disease were grouped into Progressive pathologies (Friedreich ataxia, ataxia telangiectasia, STQSM1 mutation: n. 10) and Non-Progressive (Joubert syndrome, Posterior cranial fossa tumors, ITPR1 mutation, ADCK3 mutation, cerebellar ataxia “of undetermined nature”: n. 14), according to their etiology. The ANOVA on IE and Condition (4 levels) and Progression (2 levels), as within and between factors respectively, showed no significant main effect for Progression and no significant interaction with Condition, whereas the main effect of Condition remained significant (*F*(1,66) = 3.8, *p* < 0.05, η^2^ = 0.04). Average (SD) IE for Progressive pathologies were ST = 181.8 (88.3), Anticipation DT = 206.8 (100.9), nBack DT = 224.0 (148.2), and Timing DT = 181.0 (78.2); for Non-Progressive pathologies, we saw ST = 136.4 (36.5), Anticipation DT = 179.1 (59.6), nBack DT = 193.9 (140.0), and Timing DT = 166.4 (78.5).

## Discussion

In the present study, 24 young participants affected by cerebellar pathologies (mean age 13y 1 m; 16 females) and 24 participants with typical development (mean age 13y 1 m; 14 females) were required to execute a single visuomotor precision task alone (single task condition) and with concurrent verbal anticipation, verbal timing, and n-back verbal memory tasks. Execution time and accuracy were analyzed as dependent variables combined in a single index [Inverse Efficacy, IE = RT/(1-pE)] and separately.

Results show significant main effects of Group and Condition and their significant interaction.

As expected, patients with cerebellar pathologies performed significantly worse (higher IE mean and SD values) than the age/sex-matched participants with typical development (TD) in all experimental conditions of motor control, and both for speed and accuracy.

The main effect of Condition shows that the Anticipation DT condition is the most affected dual task condition, and therefore, engaging participants in a concurrent verbal anticipation task negatively interferes with the skills of fine motor control expressed through the dominant hand, with respect to a baseline single task condition (i.e., anticipation effect). Condition nBack DT is affected as well (working memory effect), with respect to the ST condition, but that effect is due to the pathological group and not to the TD group. On the other hand, the Timing DT effect is not statistically significant.

Sensitivity and validity of the experimental protocol are supported by the results on the TD group. In particular, the anticipation effect is clear in that sample, since the Anticipation DT vs. ST comparison was the only one reaching statistical significance. Although the effect emerges from the trade-off interaction of speed and accuracy (captured by the IE index), the analyses on separated RTs and percentage of errors showed a larger contribution of execution speed. These results would therefore suggest that, as in hypotheses, engaging anticipation processing (sustained by the predictive brain system activity and including the cerebellum) would be detrimental to concurrent fine motor control in typically developed participants, by blocking the anticipation component of motor control, and forcing the use of just a feedback control mode. A concurrent verbal working memory task does not negatively interfere with motor control in the TD group, likely due to the different neuropsychological resources required in the two skills. Although timing and beat-keeping can be considered a cerebellar activity [with contribution of basal ganglia and other cortical areas depending on the task; (36, 52–55)], simultaneous timing task execution does not negatively affect motor control in our sample. A possible explanation is that cerebellar interference can be nullified by the advantage of beat-taking, which improves rather than hinders motor coordination in several clinical conditions, such as Parkinson’s disease and stuttering [e.g., (56, 57)].

How does the sample of young persons with cerebellar pathologies perform in dual task conditions? This group showed a Condition main effect as well, with significantly increased IE values in the Anticipation DT vs. ST and nBack vs. ST comparisons. Therefore, even persons with deficient cerebellar functionality perform worse for fine motor control (which is already impaired in the baseline condition) when they are contemporarily engaged in verbal anticipation processing. The amount of effect, duly weighted for the performance level, would be even larger than that shown by the TD group.

That result could suggest that the patients in our group may rely on residual anticipation skills for motor control, which would be blocked in the case of the anticipation-dual task condition. That is true for patients showing both more and less severe ataxic symptomatology (see SARA score). Moreover, in the Cerebellar group as well, execution speed could be considered a variable that is more sensitive to the observed effects with respect to accuracy.

On the other hand, an unexpected nBack effect has been found. Therefore, contrary to TD persons, patients with cerebellar pathologies overall show worse motor control when their working memory system (in verbal modality) is engaged. Nonetheless, when we analyze more patients and those with less severe symptoms separately, we find that only those patients with more severe ataxic symptoms show such a significant “working memory” effect on motor control, whereas the behavior of less severe patients does not show such an effect, as in TD participants. A first possible explanation of a “working memory” effect on motor control concerns the evidence that the cerebellum has been found to play a key role in synchronizing the activity of different cortical–subcortical networks simultaneously activated in managing dual task requests (58), or task switching (59). Hence, our patients with cerebellar dysfunction would have failed to manage the activation of multiple neural networks required in dual tasks. The absence of a timing DT effect can be ascribed, as in the TD group, to a facilitatory effect due to the “beat-taking” activity.

A further explanation is that working memory can also be affected by cerebellar pathologies, as reported by, for example, Ilg and colleagues (14), Joyce (12), and Seese (48) in a study on children, Zhang and colleagues (60). Working memory deficits are indeed included in the cerebellar symptomatology of the cognitive-affective syndrome (17, 19); cognitive tasks demanding executive functions and working memory activate bilateral cerebellar regions (5).

The impact of ataxia severity on the anticipation interference effect on motor control and the interaction with the other dual task conditions has been investigated as well. The score on the SARA scale is a significant covariate predictor in the ANOVA model on the IE dependent variable. In particular, the severity of ataxia symptoms negatively affects the anticipation effect. Anticipation effect is significant for both the sub-groups of high and low SARA scores. The higher the SARA severity, the worse the performance in the Anticipation Dual Task condition (we already underlined the increment in anticipation effect from TD to the Cerebellar group). The negative effect of the nBack task on motor control is present only for the more severe cerebellar participants and not significant for patients with a lower SARA score. No interaction between clinical severity and timing effect has been found. The data would suggest an incremental contribution of the several strategies on motor control: minimal from timing, through working memory, up to massive from the anticipation component. To our knowledge, no previous observation on similar results is available in the literature.

In order to investigate different cerebellar pathologies and their possible association with anticipation skills, patients were grouped into progressive and non-progressive ataxia. Analyses on Inverse Efficacy and in association with the four experimental conditions were carried out, but neither showed a significant effect in terms of Progression, nor have significant interactions with the different conditions been found. The results say that in our cohort of cerebellar patients, no indication for different interference effects due to the progressive or non-progressive nature of the pathology can be obtained.

According to Ghajar and Ivry (61), on-line Anticipation constitutes an automatic, rapid, and frequently non-conscious process. Strongly anchored in subcortical circuitry (specifically the cerebellum and basal ganglia), its function is to synchronize sensory cortices with the expected event during ongoing action execution. This mechanism enables behaviors such as catching a ball mid-flight without conscious trajectory calculation or ambulating over uneven terrain without visual monitoring of foot placement. The authors posit that the cerebellum plays a pivotal role in this process by generating precise temporal estimates that are subsequently relayed to the prefrontal cortex.

The present findings appear to corroborate the cerebellum’s status as a critical node for the coordination of multiple tasks [posited as a core executive function; see (62)]. In agreement with Ghajar and Ivry (60), we propose that on-line tasks involve more automatic and implicit processes, specifically Anticipation (Implicit Timing/Predictive State). This process must be functionally dissociated from voluntary planning (Explicit Timing/Cognitive Control), which we consider a higher-latency mechanism primarily subserved by fronto-parietal circuitry. The latter pertains to strategy, prospective memory (e.g., “*What I will do in 5 minutes*”), and the mental simulation of complex scenarios. Planning, conceptualized as an executive function, may developmentally rely upon the scaffolding provided by on-line anticipation (19).

Although the negative effect of the anticipation task in both groups could be explained by a general cognitive-motor load effect, the larger anticipation effect of the cerebellar cohort suggests that a fundamental aspect of such incoordination stems from a cerebellar deficit in temporal prediction and anticipation. As detailed by Ghajar and Ivry (60), attention may function as a mechanism of temporal control (in addition to its spatial role). This mechanism implicitly synchronizes neural activity to align with expected or internally generated sensory events. Such synchronization reduces variability in neural processing, thereby enhancing perception and action.

### Limitations and further research

As expected, the Cerebellar group showed lower cognitive and attentional levels, and that may have increased error variance. Nonetheless, comparison among the four conditions within each patient minimizes such a difference. A second control group composed of acquired brain injury patients with equal IQ and/or attention skills with respect to the Cerebellar group would be useful in further research to understand the impact of general cognitive and attentional level. A specific study analyzing separately progressive and non-progressive cerebellar pathologies, or sites and width of cortical and subcortical lesions, would help achieve a better understanding of the cortical–subcortical circuitry related to anticipatory motor control.

The Cerebellar group was heterogeneous in diagnosis, but the number of cases for each pathology did not allow for differential analyses. Further studies about possible differential effects for pathologies are needed. Due to such heterogeneity and the clinical characteristics of the cerebellar pathology, the dependent variable variance was larger for the cerebellar group compared to the control group. Nevertheless, intra-group comparisons have been executed and discussed. Less heterogeneous samples should be selected for future research if possible.

Finally, it should be noted that while the motor task (NEPSY-II, 46) is a standardized measure with established reliability, the cognitive tasks relied on *ad hoc* built experimental paradigms. Consequently, standardized reliability indices (such as test–retest or internal consistency) for these specific versions in the clinical population are not available in the current literature. This may limit the generalizability of our results, and future studies should aim to confirm these findings using validated clinical tools.

## Conclusion

The results showed that the dual-task condition negatively affects fine motor control of the upper limb in the two groups. A significant negative effect of the anticipation task was found for both the cerebellar and control groups. The results are consistent with the models describing the cerebellum as a key hub in anticipatory control. Anticipatory control is a damaged component in the control deficit of cerebellar patients. Finally, the cerebellar role in synchronizing the different cerebral networks involved in dual-task processing is strengthened.

This study underlines the crucial role of anticipatory and feedforward mechanisms in motor control of both typically developed children and those with cerebellar pathologies and defines the importance of predictive neuropsychological skills in cognitive and motor fluidity. In cerebellar pathologies rehabilitation, the strengthening of such skills [e.g., mirror therapy, action observation treatment, motor therapy, quiet eye training, *etc*., (63)], together with traditional motor therapy, would support the acquisition of new and more functional schemas of movement, slow down cognitive decline, and increase motor, cognitive, and behavioral control.

## Data Availability

The raw data supporting the conclusions of this article will be made available by the authors, without undue reservation.

## References

[1] Benussi A Batsikadze G França C Cury RG Maas RPPWM. The therapeutic potential of non-invasive and invasive cerebellar stimulation techniques in hereditary ataxias. Cells. (2023) 12:1193. doi: 10.3390/cells12081193, 37190102 PMC10137097

[2] Coarelli G Wirth T Tranchant C Koenig M Durr A Anheim M. The inherited cerebellar ataxias: an update. J Neurol. (2023) 270:208–22. doi: 10.1007/s00415-022-11383-6, 36152050 PMC9510384

[3] Cunha P Petit E Coutelier M Coarelli G Mariotti C Faber J . Extreme phenotypic heterogeneity in non-expansion spinocerebellar ataxias. Am J Hum Genet. (2023) 110:1098–109. doi: 10.1016/j.ajhg.2023.05.009, 37301203 PMC10357418

[4] Salari M Etemadifar M Rashedi R Mardani. A review of ocular movement abnormalities in hereditary cerebellar ataxias. Cerebellum. (2023) 23:702–21. doi: 10.1007/s12311-023-01554-0, 37000369

[5] Schmahmann JD. The cerebellum and cognition. Neurosci Lett. (2019) 688:62–75. doi: 10.1016/j.neulet.2018.07.005, 29997061

[6] Schmitz-Hübsch T du Montcel ST Baliko L Berciano J Boesch S Depondt C . Scale for the assessment and rating of ataxia. Neurology. (2006) 66:1717–20. doi: 10.1212/01.wnl.0000219042.60538.92 16769946

[7] Sullivan R Yau WY O’Connor E Houlden H. Spinocerebellar ataxia: an update. J Neurol. (2019) 266:533–44. doi: 10.1007/s00415-018-9076-4, 30284037 PMC6373366

[8] Manto M Gandini J Feil K Strupp M. Cerebellar ataxias: an update. Curr Opin Neurol. (2020) 33:150–60. doi: 10.1097/WCO.0000000000000774, 31789706

[9] Marmolino D Manto M. Past, present and future therapeutics for cerebellar ataxias. Curr Neuropharmacol. (2010) 8:41–61. doi: 10.2174/157015910790909476, 20808545 PMC2866461

[10] Buesch K Zhang R. A systematic review of disease prevalence, health-related quality of life, and economic outcomes associated with Friedreich’s Ataxia. Curr Med Res Opin. (2022) 38:1739–49. doi: 10.1080/03007995.2022.2112870, 35983717

[11] Curie A Lion-François L Valayannopoulos V Perreton N Gavanon M Touil N . Clinical characteristics, developmental trajectory, and caregiver burden of patients with Creatine transporter deficiency (SLC6A8). Neurology. (2024) 102:e209243. doi: 10.1212/WNL.0000000000209243, 38531017

[12] Joyce MR Nadkarni PA Kronemer SI Margron MJ Slapik MB Morgan OP . Quality of life changes following the onset of cerebellar Ataxia: symptoms and concerns self-reported by Ataxia patients and informants. Cerebellum. (2022) 21:592–605. doi: 10.1007/s12311-022-01393-5, 35334077

[13] Tran AM Jnah AJ De Castro Pretelt MJ. Genetics review: Joubert syndrome. Neonatal Netw. (2025) 44:159–66. doi: 10.1891/NN-2024-0052, 40537162

[14] Manto M Bower JM Conforto AB Delgado-García JM da Guarda SNF Gerwig M . Consensus paper: roles of the cerebellum in motor control—the diversity of ideas on cerebellar involvement in movement. Cerebellum. (2012) 11:457–87. doi: 10.1007/s12311-011-0331-9, 22161499 PMC4347949

[15] Olivito G Lupo M Iacobacci C Clausi S Romano S Masciullo M . Structural cerebellar correlates of cognitive functions in spinocerebellar ataxia type 2. J Neurol. (2018) 265:597–606. doi: 10.1007/s00415-018-8738-6, 29356974

[16] Riva. The cerebellum contributes to higher functions during development: evidence from a series of children surgically treated for posterior fossa tumours. Brain. (2000) 123:1051–61. doi: 10.1093/brain/123.5.1051, 10775549

[17] Schmahmann JD. Disorders of the cerebellum: Ataxia, dysmetria of thought, and the cerebellar cognitive affective syndrome. J Neuropsychiatry Clin Neurosci. (2004) 16:367–78. doi: 10.1176/jnp.16.3.367, 15377747

[18] Guell X Gabrieli JDE Schmahmann JD. Embodied cognition and the cerebellum: perspectives from the Dysmetria of thought and the universal cerebellar transform theories. Cortex. (2018) 100:140–8. doi: 10.1016/j.cortex.2017.07.005, 28779872

[19] Schmahmann JD Guell X Stoodley CJ Halko MA. The theory and neuroscience of cerebellar cognition. Annu Rev Neurosci. (2019) 42:337–64. doi: 10.1146/annurev-neuro-070918-050258, 30939101

[20] Kawato M Kuroda S Schweighofer N. Cerebellar supervised learning revisited: biophysical modeling and degrees-of-freedom control. Curr Opin Neurobiol. (2011) 21:791–800. doi: 10.1016/j.conb.2011.05.014, 21665461

[21] Caligiore D Pezzulo G Baldassarre G Bostan AC Strick PL . Consensus paper: towards a systems-level view of cerebellar function: the interplay between cerebellum, basal ganglia, and cortex. Cerebellum. (2017) 16:203–29. doi: 10.1007/s12311-016-0763-3, 26873754 PMC5243918

[22] Leggio M Molinari M. Cerebellar sequencing: a trick for predicting the future. Cerebellum. (2015) 14:35–8. doi: 10.1007/s12311-014-0616-x, 25331541

[23] Patla AE. Strategies for dynamic stability during adaptive human locomotion. IEEE Eng Med Biol Mag. (2003) 22:48–52. doi: 10.1109/MEMB.2003.1195695, 12733458

[24] Olivito G Brunamonti E Clausi S Pani P Chiricozzi FR Giamundo M . Atrophic degeneration of cerebellum impairs both the reactive and the proactive control of movement in the stop signal paradigm. Exp Brain Res. (2017) 235:2971–81. doi: 10.1007/s00221-017-5027-z, 28717819

[25] Ghajar J Ivry RB Cognitive and Neurobiological Research Consortium. The predictive brain state: timing deficiency in traumatic brain injury. Neurorehabil Neural Repair. (2008) 22:217–27. doi: 10.1177/1545968308315600, 18460693 PMC4338277

[26] Wolpert DM Ghahramani Z Jordan MI. An internal model for sensorimotor integration. Science. (1995) 269:1880–2. doi: 10.1126/science.7569931, 7569931

[27] Ramnani N Miall RC. A system in the human brain for predicting the actions of others. Nat Neurosci. (2004) 7:85–90. doi: 10.1038/nn1168, 14699420

[28] Kühn S Seurinck R Fias W Waszak F. The internal anticipation of sensory action effects: when action induces FFA and PPA activity. Front Hum Neurosci. (2010) 4:54. doi: 10.3389/fnhum.2010.00054, 20661462 PMC2907885

[29] Vaziri S Diedrichsen J Shadmehr R. Why does the brain predict sensory consequences of oculomotor commands? Optimal integration of the predicted and the actual sensory feedback. J Neurosci. (2006) 26:4188–97. doi: 10.1523/JNEUROSCI.4747-05.2006, 16624939 PMC1473981

[30] Donnarumma F Costantini M Ambrosini E Friston K Pezzulo G. Action perception as hypothesis testing. Cortex. (2017) 89:45–60. doi: 10.1016/j.cortex.2017.01.016, 28226255 PMC5383736

[31] Butti N Corti C Finisguerra A Bardoni A Borgatti R Poggi G . Cerebellar damage affects contextual priors for action prediction in patients with childhood brain tumor. Cerebellum. (2020) 19:799–811. doi: 10.1007/s12311-020-01168-w, 32699945

[32] Urgesi C Butti N Finisguerra A Biffi E Valente EM Romaniello R . Social prediction in pediatric patients with congenital, non-progressive malformations of the cerebellum: from deficits in predicting movements to rehabilitation in virtual reality. Cortex. (2021) 144:82–98. doi: 10.1016/j.cortex.2021.08.008, 34662720

[33] Oldrati V Butti N Ferrari E Strazzer S Romaniello R Borgatti R . Neurorestorative effects of cerebellar transcranial direct current stimulation on social prediction of adolescents and young adults with congenital cerebellar malformations. Neuroimage Clin. (2024) 41:103582. doi: 10.1016/j.nicl.2024.103582, 38428326 PMC10944181

[34] Ragland JD Turetsky BI Gur RC Gunning-Dixon F Turner T Schroeder L . Working memory for complex figures: an fMRI comparison of letter and fractal n-back tasks. Neuropsychology. (2002) 16:370–9. doi: 10.1037/0894-4105.16.3.370, 12146684 PMC4332798

[35] Drobyshevsky A Baumann SB Schneider W. A rapid fMRI task battery for mapping of visual, motor, cognitive, and emotional function. NeuroImage. (2006) 31:732–44. doi: 10.1016/j.neuroimage.2005.12.016, 16488627 PMC1620013

[36] Ivry RB Spencer RM Zelaznik HN Diedrichsen J. The cerebellum and event timing. Ann N Y Acad Sci. (2002) 978:302–17. doi: 10.1111/j.1749-6632.2002.tb07576.x, 12582062

[37] Ivry RB Spencer RM. The neural representation of time. The neural representation of time. Curr Opin Neurobiol. (2004) 14:225–32. doi: 10.1016/j.conb.2004.03.013, 15082329

[38] Lungu OV Bares M Liu T Gomez CM Cechova I Ashe J. Trial-to-trial adaptation: parsing out the roles of cerebellum and BG in predictive motor timing. J Cogn Neurosci. (2016) 28:920–34. doi: 10.1162/jocn_a_00943, 26942317

[39] Nozaradan S Schwartze M Obermeier C Kotz SA. Specific contributions of basal ganglia and cerebellum to the neural tracking of rhythm. Cortex. (2017) 95:156–68. doi: 10.1016/j.cortex.2017.08.015, 28910668

[40] Roid GH Koch. "Leiter-3: nonverbal cognitive and neuropsychological assessment". In: Handbook of Nonverbal Assessment. London: Springer International Publishing (2017). p. 127–50.

[41] Raven JC. "CPM Raven’s coloured progressive matrices". In: PsycTESTS Dataset. Washington DC: APA PsycTests (2012).

[42] Belacchi C Scalisi T Cannoni E Cornoldi C. CPM – Coloured Progressive Matrices. Standardizzazione Italiana. Manuale. Florence: Giunti Psychometrics (2008).

[43] Picone S Orsini A Pezzuti L. SPM: Standard Progressive Matrices. Standardizzazione Italiana. Florence: Giunti Psychometrics (2018).

[44] Wechsler D. "Wechsler intelligence scale for children, fourth edition". In: Orsini A Pezzuti L Picone L, editors. WISC – IV: Contributo Alla Taratura Italiana. Florence: Giunti OS PsycTESTS Dataset (2012)

[45] Wechsler D. "Wechsler preschool and primary scale of intelligence--third edition". In: Orsini A Pezzuti L Picone L, editors. WISC – IV: Contributo alla taratura italiana. Florence: Giunti OS, 2008 PsycTESTS Dataset (2012)

[46] Korkman M Kirk U Kemp S. "NEPSY - Second edition". In: PsycTESTS Dataset NEPSY - Second Edition. San Antonio, TX: PsycTESTS Dataset (2012)

[47] Urgesi C Franco. NEPSY-II. Batteria per la valutazione neuropsicologica dello sviluppo - Adattamento Italiano. Florence: Giunti O.S (2011).

[48] Psychology Software Tools, Inc. E-prime 3.0 [Software]. Sharpsburg (PA): Psychology Software Tools (2016).

[49] Burgess PW Shallice T. The Hayling and Brixton Tests. Bury St Edmunds UK: Thames Valley Test Company (1997)

[50] Murphy FC Klein RM. The effects of nicotine on spatial and non-spatial expectancies in a covert orienting task. Neuropsychologia. (1998) 36:1103–14. doi: 10.1016/S0028-3932(98)00012-8, 9842757

[51] Townsend JT Ashby. The Stochastic Modeling of Elementary Psychological Processes. Cambridge: Cambridge University Press (1983).

[52] Salman MS. Topical review: the cerebellum: it’s about time! But timing is not everything-new insights into the role of the cerebellum in timing motor and cognitive tasks. J Child Neurol. (2002) 17:1–9. doi: 10.1177/088307380201700101, 11913561

[53] Baumann O Borra RJ Bower JM Cullen KE Habas C Ivry RB . Consensus paper: the role of the cerebellum in perceptual processes. Cerebellum. (2015) 14:197–220. doi: 10.1007/s12311-014-0627-7, 25479821 PMC4346664

[54] Van Der Steen MC Schwartze M Kotz SA Keller PE. Modeling effects of cerebellar and basal ganglia lesions on adaptation and anticipation during sensorimotor synchronization. Ann N Y Acad Sci. (2015) 1337:101–10. doi: 10.1111/nyas.12628, 25773623

[55] Arleo A Bareš M Bernard JA Bogoian HR Bruchhage MMK Bryant P . Consensus paper: cerebellum and ageing. Cerebellum. (2023) 23:802–32. doi: 10.1007/s12311-023-01577-7, 37428408 PMC10776824

[56] Huang X Dong K Gan C Xu Z Lei D Dong X . Effect of rhythmically cued exercise interventions on functions in patients with Parkinson disease: a meta-analysis. Phys Ther. (2024) 104:pzad158. doi: 10.1093/ptj/pzad158, 37962936

[57] Frankford SA Heller Murray ES Masapollo M Cai S Tourville JA Nieto-Castañón A . The neural circuitry underlying the “rhythm effect” in stuttering. J Speech Lang Hear Res. (2021) 64:2325–46. doi: 10.1044/2021_JSLHR-20-00328, 33887150 PMC8740675

[58] Wu T Liu J Hallett M Zheng Z Chan P. Cerebellum and integration of neural networks in dual-task processing. NeuroImage. (2013) 65:466–75. doi: 10.1016/j.neuroimage.2012.10.004, 23063842 PMC4173076

[59] Berger A Sadeh M Tzur G Shuper A Kornreich L Inbar D . Task switching after cerebellar damage. Neuropsychology. (2005) 19:362–70. doi: 10.1037/0894-4105.19.3.362, 15910122

[60] Zhang P Duan L Ou Y Ling Q Cao L Qian H . The cerebellum and cognitive neural networks. Front Hum Neurosci. (2023) 17:17. doi: 10.3389/fnhum.2023.1197459, 37576472 PMC10416251

[61] Ghajar J Ivry RB. The predictive brain state: asynchrony in disorders of attention? Neuroscientist. (2009) 15:232–42. doi: 10.1177/1073858408326429, 19074688 PMC4342364

[62] Miyake A Friedman NP Emerson MJ Witzki AH Howerter A Wager TD. The Unity and Diversity of executive functions and their contributions to complex “frontal lobe” tasks: a latent variable analysis. Cogn Psychol. (2000) 41:49–100. doi: 10.1006/cogp.1999.0734, 10945922

[63] Miles CAL Wood G Vine SJ Vickers JN Wilson MR. Quiet eye training facilitates visuomotor coordination in children with developmental coordination disorder. Res Dev Disabil. (2015) 40:31–41. doi: 10.1016/j.ridd.2015.01.005, 25721344

