# Supplementary material for: Anticipation strategies of motor control in children and adolescents with cerebellar pathologies and typical development: a dual task paradigm
Source: Front Neurol. 2026 Jun 24;17:1830073. doi: 10.3389/fneur.2026.1830073 (PMC13341546; doi:10.3389/fneur.2026.1830073)
Supplement: Supplementary file 1 [file Table_1.DOCX]

APPENDIX


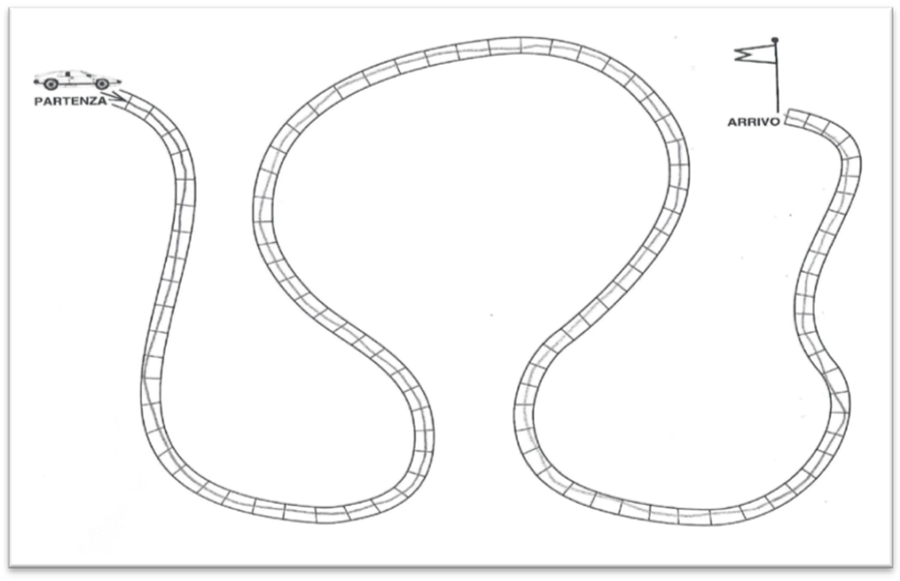


*Figure A1. Performance of a representative participant from the TD group in the ST condition.*


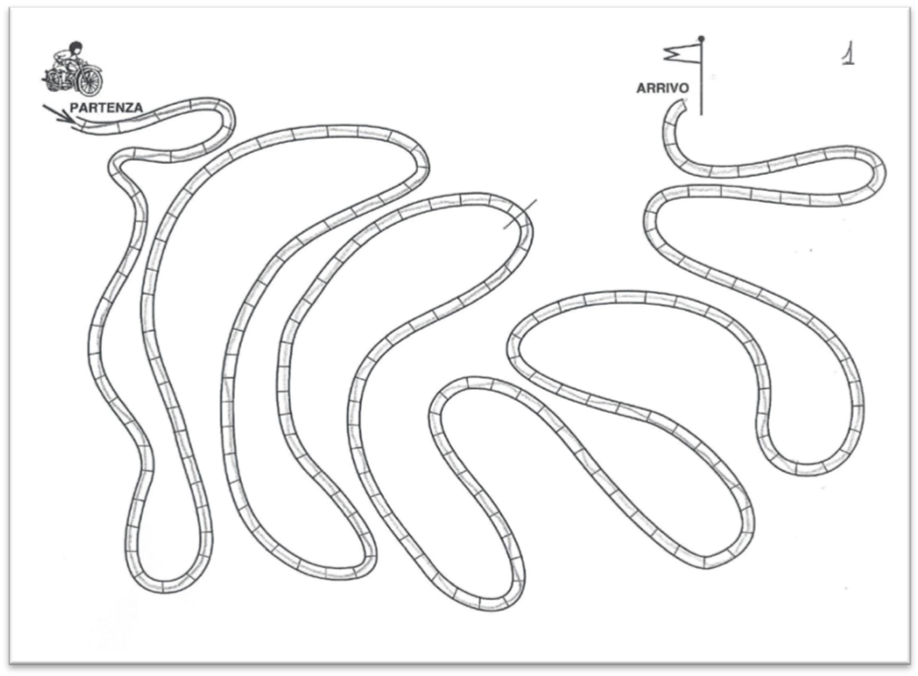


*Figure A2. Performance of a representative participant from the TD group in the ST condition.*


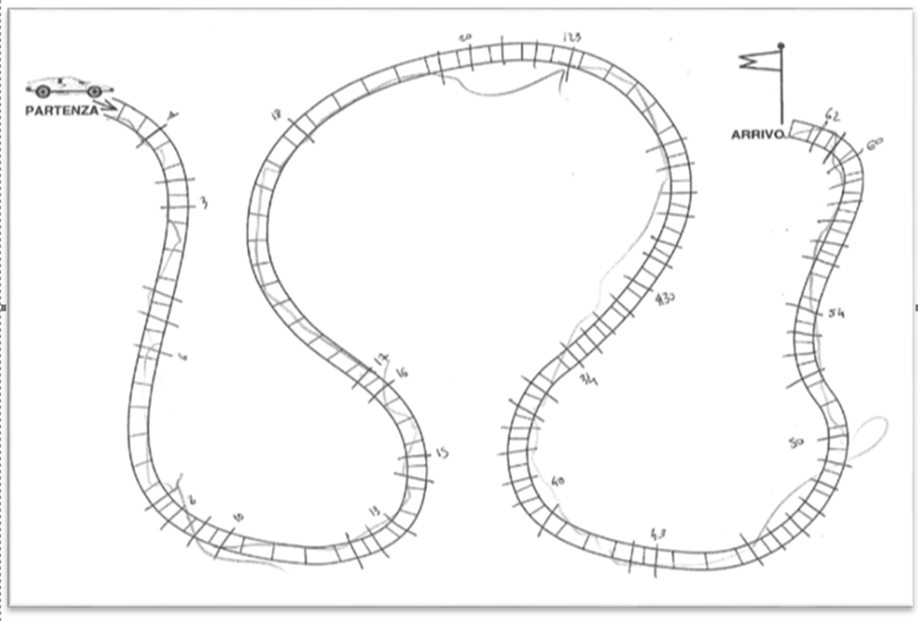


*Figure A3. Performance of a representative participant from the Cerebellar group in the ST condition.*


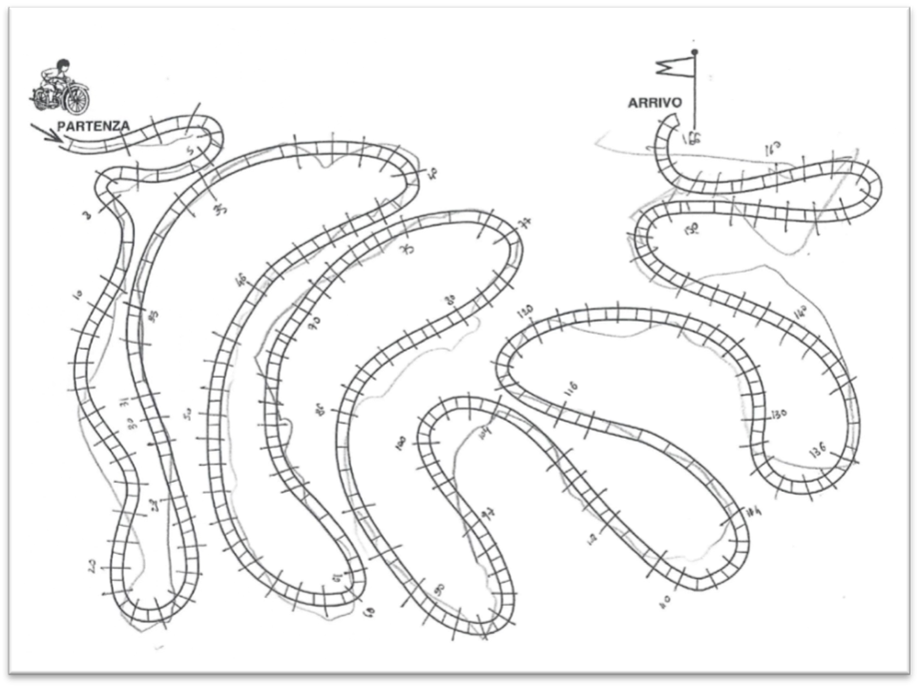


*Figure A4. Performance of a representative participant from the Cerebellar group in the ST condition.*
